# Supplementary material for: Pervasive coexpression of spatially proximal genes is buffered at the protein level
Source: Mol Syst Biol. 2017 Aug 23;13(8):937. doi: 10.15252/msb.20177548 (PMC5572396; doi:10.15252/msb.20177548)
Supplement: Supplementary file 1 — Appendix [file MSB-13-937-s001.pdf]

Appendix

Pervasive co-expression of spatially proximal genes is buffered at the protein level

Kustatscher, Grabowski and Rappsilber

Table of contents

Appendix Fig S1. Characterization of the 4,188 genes in the LCL dataset as part of the core proteome 2

Appendix Fig S2. Individual human chromosomes have distinct co-regulation curves 3

Appendix Fig S3. Determining the optimal number of clusters for k-means clustering 4

Appendix Fig S4. Protein co-regulation clusters are more clearly associated with distinct biological processes than mRNA clusters 5

Appendix Table S1. Co-regulation of bidirectional gene pairs without shared function is buffered at the protein level 6

Appendix Table S2. Correlation coefficients between mRNA co-regulation or protein co-regulation with Hi-C contacts, by chromosome 7

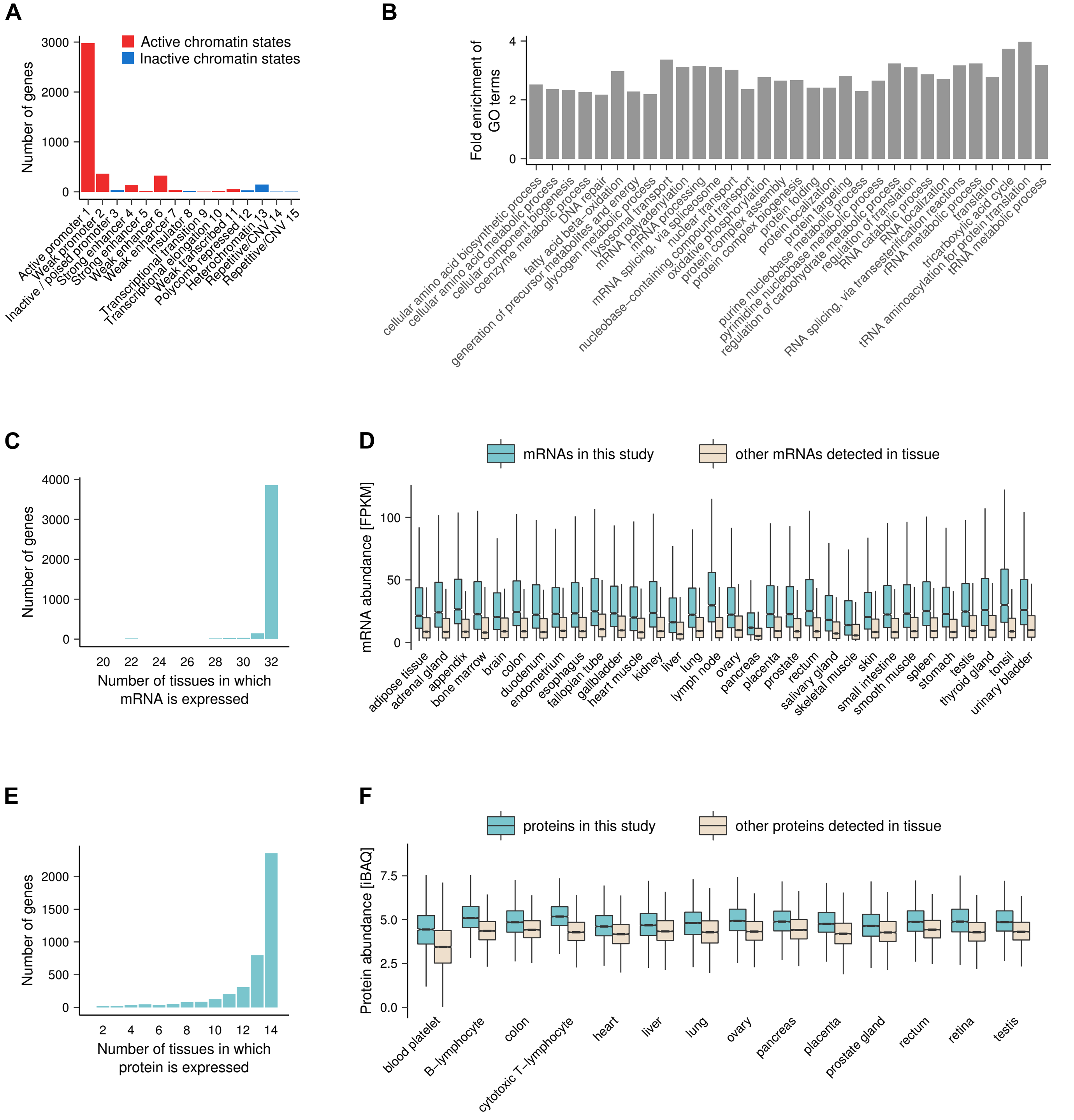

Appendix Fig S1. Characterization of the 4,188 genes in the LCL dataset as part of the core proteome.

A The transcription start sites of most of these genes map to regions in an "active promoter" chromatin state. This chromatin state is characterised by high levels of activating histone modifications such as H3K4me3 and H3K9ac (Ernst *et al*, 2011).

B Compared to the whole genome, these genes are enriched for GO terms indicating ubiquitous biological processes, as assessed using the Panther classification system (Mi *et al*, 2016).

C, D The mRNAs of the genes in this study are expressed in all human tissues for which mRNA expression has been measured (Uhlén *et al*, 2015) and are also more abundant than other mRNAs expressed in each tissue.

E, F The proteins of these genes are detected in most human tissues for which reasonable proteome coverage is available (Wilhelm *et al*, 2014) and these proteins are also more abundant than other proteins detected in each tissue.

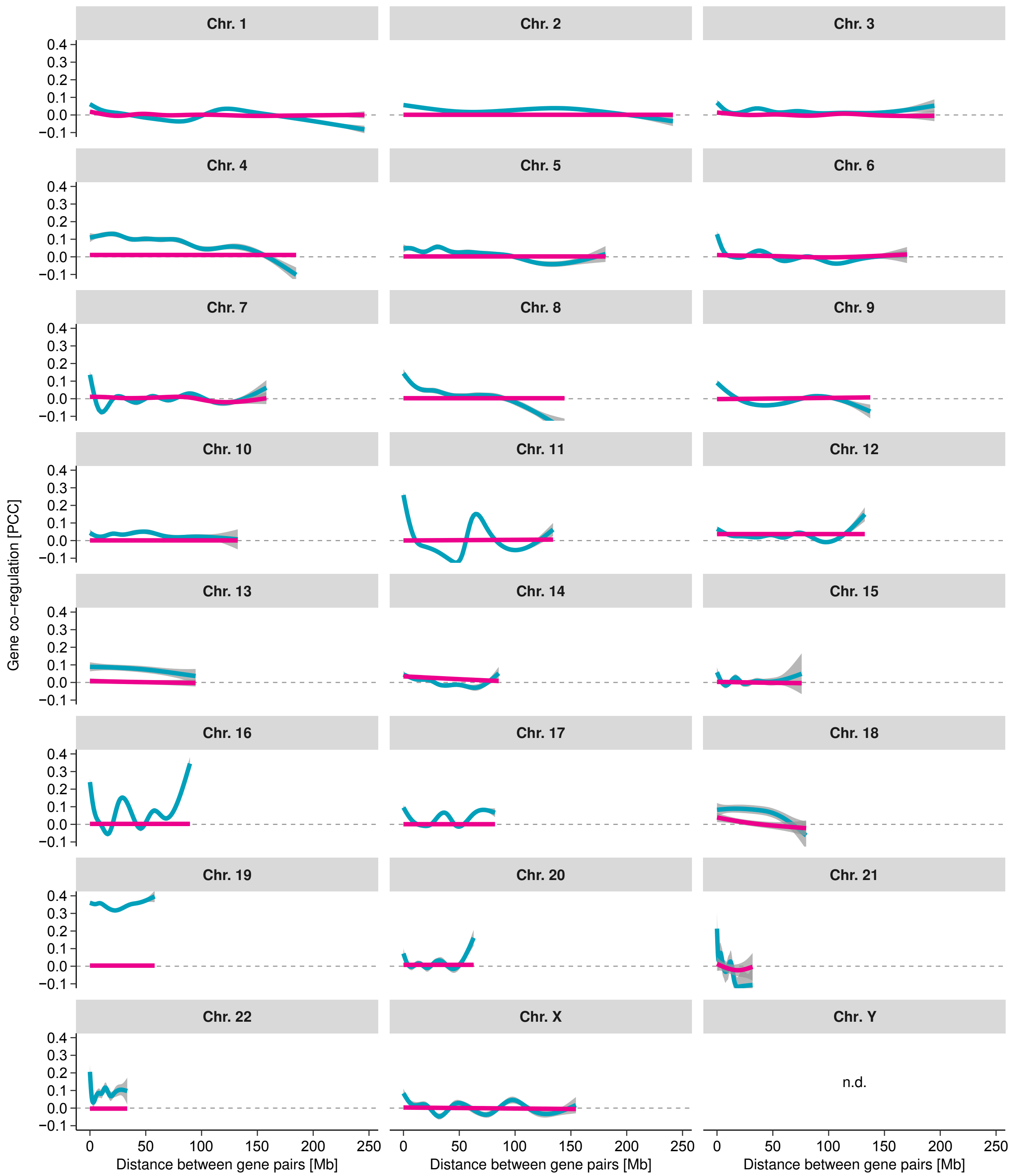

Appendix Fig S2. Individual human chromosomes have distinct co-regulation curves.

The relationship between gene distance and co-regulation across LCLs is different on every chromosome. The co-regulation strength often increases at short distances. Multiple peaks in intervals of tens of megabases are also common, suggesting that co-regulation may be connected to long-range chromosome folding, which may bring gene pairs into close 3D proximity. Curves represent a generalized additive model fitted to data from all gene pairs.

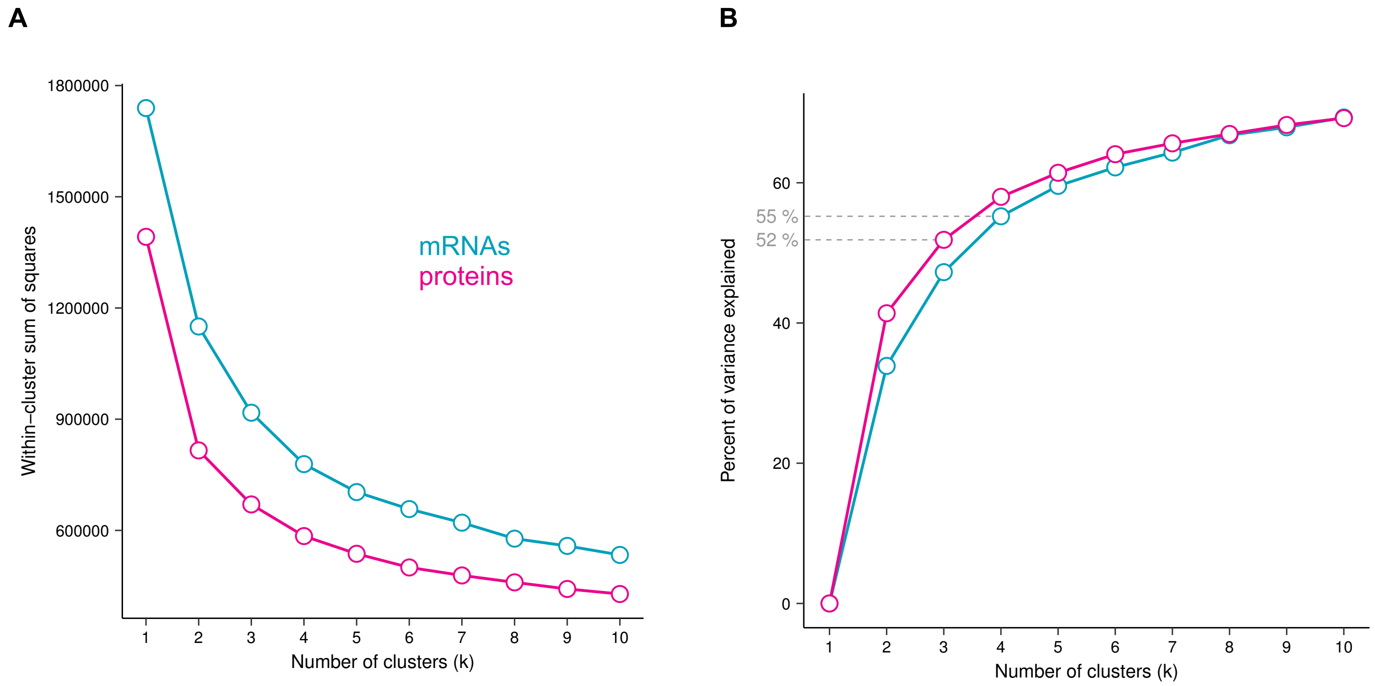

Appendix Fig S3. Determining the optimal number of clusters for k-means clustering.

A *k*-means clustering requires that the number of clusters to be detected is specified in advance. The optimal number of clusters (*k*) was determined using the Elbow method. Although a clear "elbow" could not be determined, the within-cluster sum of squares only decreases sharply until about 4 mRNA or 3 protein clusters.

B With these values of *k* more than 50% variance is explained for both mRNA and protein co-regulation.

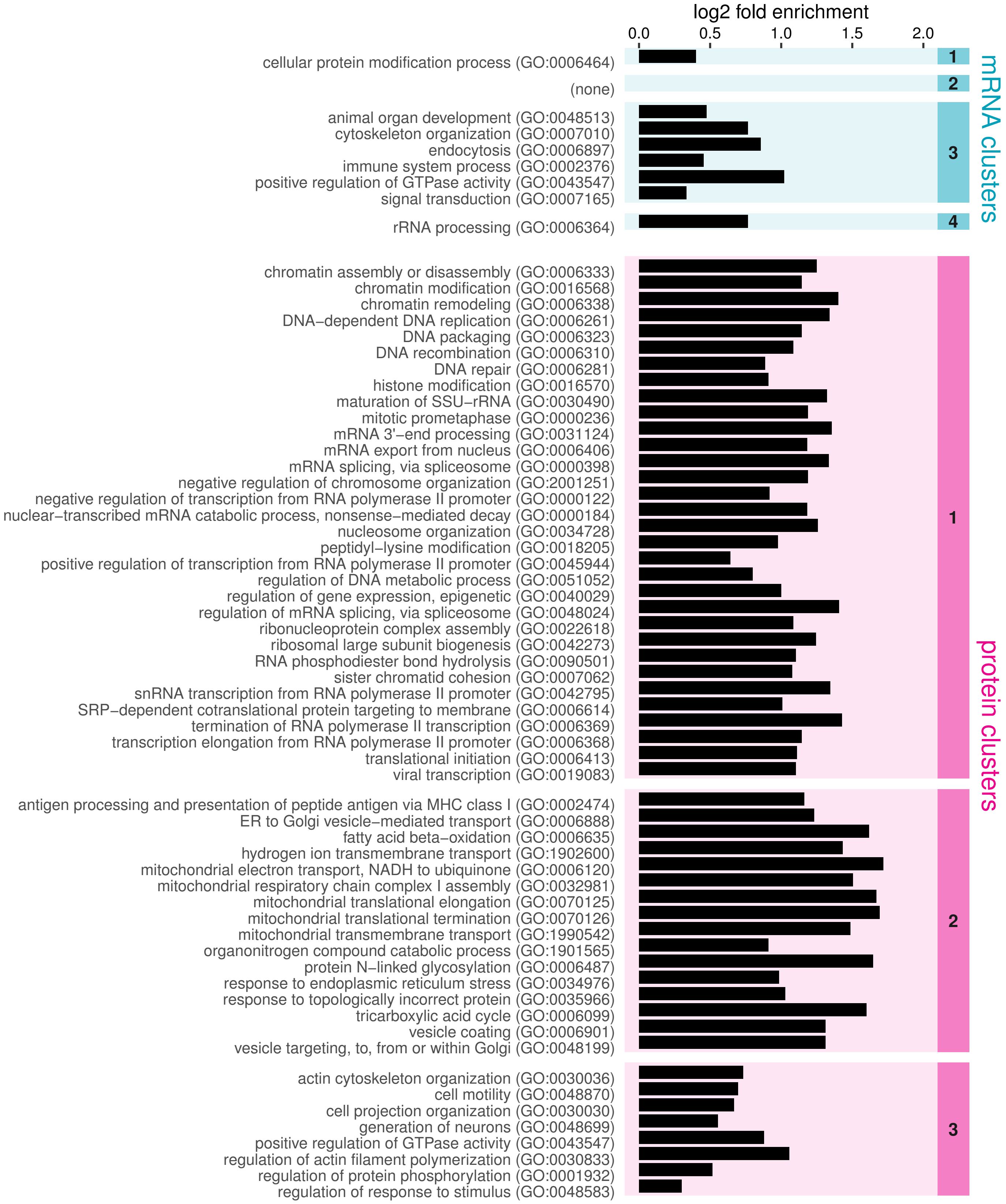

Appendix Fig S4. Protein co-regulation clusters are more clearly associated with distinct biological processes than mRNA clusters.

Shown are Gene Ontology biological processes that are significantly enriched in each co-regulation cluster ($P < 0.05$ after Bonferroni correction). Only the most specific GO terms are shown here, any enriched parent terms where omitted for clarity. The analysis was done using the Panther classification system (Mi *et al*, 2016).

Appendix Table S1. Co-regulation of bidirectional gene pairs without shared function is buffered at the protein level.

These 31 gene pairs are transcribed from bidirectional promoters and have significantly co-regulated mRNA abundances (Pearson's correlation coefficient, PCC > 0.5, BH adj. *P* value < 0.001). For most gene pairs, protein co-regulation is either attenuated (weaker, but still significant) or buffered (not significant). Literature searches revealed that these gene pairs do not generally have shared functions, in contrast to the few gene pairs whose co-regulation is sustained at the protein level.

Gene 1	Gene 2	Protein 1	Protein 2	mRNA PCC	Protein PCC	Protein co-regulation	Common function	Functional annotation (manual literature evaluation)
PARP9	DTX3L	Q8IXQ6	Q8TDB6	0.84	0.96	sustained	Yes	The two proteins interact and together play a role in PARP1-dependent DNA damage repair
MPST	TST	P25325	Q16762	0.68	0.84	sustained	Yes	Both proteins are sulfurtransferases involved, among other things, in cyanide detoxification
RNMT	FAM210A	O43148	Q96ND0	0.83	0.57	attenuated	No	RNMT is a nuclear mRNA capping factor, FAM210A an uncharacterised mitochondrial protein
SSNA1	ANAPC2	O43805	Q9UJX6	0.71	0.55	buffered	No	SSNA1 is a centrosomal protein, ANAPC2 is a subunit of the anaphase promoting complex
MRPS12	SARS2	O15235	Q9NP81	0.50	0.50	sustained	Yes	Both proteins are involved in mitochondrial protein translation
SEC61B	ALG2	P60468	Q9H553	0.51	0.49	buffered	Yes	ALG2 functions in protein glycosylation at cytosolic side of ER, SEC61B in protein translocation into ER
CUL4A	PCID2	Q13619	Q5JVF3	0.62	0.40	buffered	No	PCID2 participates in nuclear mRNA export, CUL4A is ubiquitin ligase in DNA repair, replication, etc.
SAE1	ZC3H4	Q9UBE0	Q9UPT8	0.57	0.39	buffered		NA (ZC3H4 is uncharacterized)
MVK	MMAB	Q03426	Q96EY8	0.62	0.37	buffered	No	Synthesis of cytoplasmic isopentenyl diphosphate (MVK) & mitochondrial adenosylcobalamin (MMAB)
DBI	C2orf76	P07108	Q3KRA6	0.63	0.37	buffered		NA (C2orf76 is uncharacterized)
HRSP12	POP1	P52758	Q99575	0.54	0.34	buffered	No	HRSP12 is cytoplasmic ribonuclease for mRNA, POP1 is nuclear ribonuclease for tRNA
TCEB1	TMEM70	Q15369	Q9BUB7	0.57	0.33	buffered	No	TCEB1 is a nuclear transcription factor, TMEM70 a mitochondrial transmembrane protein
HOOK3	RNF170	Q86VS8	Q96K19	0.54	0.31	buffered	No	HOOK3 is Golgi-based microtubule-binding protein, RNF170 is ER-membrane-based E3 ubiquitin ligase
UCHL3	COMMD6	P15374	Q7Z4G1	0.69	0.21	buffered		NA (proteins not sufficiently characterized)
PCMT1	NUP43	P22061	Q8NFH3	0.64	0.15	buffered	No	NUP43 belongs to nuclear pore complex, PCMT1 is involved in repair of damaged cytoplasmic proteins
SKIV2L2	DHX29	P42285	Q7Z478	0.54	0.14	buffered	No	Nucleolar SKIV2L2: pre-mRNA & rRNA processing, cytoplasmic DHX29: helicase for translation initiation
DDX51	NOC4L	Q8N8A6	Q9BVI4	0.60	0.10	buffered	Unclear	NOC4L is uncharacterised nucleolar protein, DDX51 is nucleolar helicase for rRNA processing
TSN	NIFK	Q15631	Q9BYG3	0.52	0.09	buffered	No	Translin involved in pre-miRNA degradation, NIFK is nucleolar protein involved in ribosome biogenesis
BST2	MVB12A	Q10589	Q96EY5	0.54	0.07	buffered	Unclear	Both affect retrovirus budding from cell membrane, but have additional non-overlapping functions
COPE	DDX49	O14579	Q9Y6V7	0.63	-0.02	buffered	No	COPE is cytosolic protein coating transport vesicles, DDX49 a probable nucleolar RNA helicase
BUD31	PDAP1	P41223	Q13442	0.52	-0.05	buffered		NA (PDAP1 is uncharacterized)
SH3BGRL	HMGN5	O75368	P82970	0.54	-0.10	buffered	No	HMGN5: regulates chromatin compaction, SH3BGRL: nuclear/cytoplasmic thioredoxin protein
DRAP1	C11orf68	Q14919	Q9H3H3	0.53	-0.11	buffered		NA (C11orf68 is uncharacterized)
NUDCD1	ENY2	Q96RS6	Q9NPA8	0.52	-0.13	buffered		NA (NUDCD1 is uncharacterized)
GTF2H1	HPS5	P32780	Q9UPZ3	0.53	-0.16	buffered	No	GTF2H1 is transcription factor, HPS5 a membrane protein for lysosome-related organelles biogenesis
DFFA	PEX14	O00273	O75381	0.53	-0.23	buffered	No	PEX14: membrane protein for peroxisomal import, DFFA: regulates apoptotic DNA fragmentation
CDC45	UFD1L	O75419	Q92890	0.53	-0.31	buffered	No	UFD1L: proteasomal ubiquitin-dependent protein degradation, CDC45: DNA replication factor
CDC123	NUDT5	O75794	Q9UKK9	0.65	-0.34	buffered	No	NUDT5 is nuclear metabolic enzyme, CDC123 is cytoplasmic regulator of translation.
HTRA2	AUP1	O43464	Q9Y679	0.67	-0.34	buffered	No	AUP1: translocation of misfolded proteins from ER lumen to cytoplasm, HTRA2: mitochondrial protease
ATP6V1A	NAA50	P38606	Q9GZZ1	0.62	-0.37	buffered	No	ATP6V1A is subunit of a vacuolar ATPase, NAA50 is cytoplasmic protein acetyltransferase
EMG1	PHB2	Q92979	Q99623	0.66	-0.43	buffered	No	PHB2: respiration in mitochondrial membrane (mainly), EMG1: nucleolar rRNA biosynthesis

Protein co-regulation: sustained / attenuated / buffered

Common function: Yes / Unclear / No

Chromosome	mRNA - HiC correlation	mRNA - HiC *P value*	protein - HiC correlation	protein - HiC *P value*	Number of gene pairs
1	0.055	6.40E-66	0.016	4.40E-07	98,807
2	0.05	8.30E-24	0.002	0.72	40,914
3	0.067	1.10E-29	0.01	0.11	28,538
4	0.076	4.40E-15	-0.011	0.24	10,655
5	0.028	0.00019	0.009	0.24	17,401
6	0.132	2.00E-83	0.022	0.0012	21,193
7	0.096	4.60E-36	0.013	0.086	17,039
8	0.118	2.20E-34	0.006	0.52	10,580
9	0.112	1.50E-34	0.001	0.89	11,892
10	0.012	0.14	0.01	0.26	14,012
11	0.21	1.89E-318	0.005	0.4	32,127
12	0.057	1.50E-20	0.003	0.62	26,100
13	0.018	0.39	0.031	0.15	2,145
14	0.041	3.60E-06	0.018	0.046	13,041
15	0.035	0.0016	0.006	0.59	8,249
16	0.154	1.00E-85	0.011	0.16	16,110
17	0.041	8.10E-13	0.005	0.42	30,114
18	0.016	0.55	0.056	0.03	1,485
19	0.017	0.0018	-0.007	0.21	33,927
20	0.038	0.0024	-0.006	0.66	6,216
21	0.192	2.90E-07	0.061	0.11	703
22	0.046	0.0024	0.002	0.9	4,362
X	0.088	5.70E-18	0.007	0.49	9,686
all intrachromosomal gene pairs	0.117	0	0.008	4.10E-07	455,296
all interchromosomal gene pairs	0.107	0	0.002	1.70E-09	7,915,477

Appendix Table S2. Correlation coefficients between mRNA co-regulation or protein co-regulation with Hi-C contacts, by chromosome

Pearson's correlation coefficients between mRNA co-regulation or protein co-regulation maps with the number of log2 Hi-C contacts were calculated for every chromosome. These numbers are also indicated in grey insets in Fig 1F and Fig EV3. The overall correlation between mRNA co-regulation or protein co-regulation and Hi-C contacts is also reported. Most chromosome show a highly significant correlation between gene co-regulation and Hi-C contacts, but only at the mRNA level. Note that the significance of a correlation (see *P values*) depends on the number of gene pairs that were tested. For large test sets even a very small correlation coefficient can be statistically significant, although this may not necessarily be biologically relevant. For example, see the 98,807 gene pairs on chromosome 1 with a protein - HiC correlation of 0.016 (*P* ~ 4.4E-7).
