# Supplementary material for: Pervasive coexpression of spatially proximal genes is buffered at the protein level
Source: Mol Syst Biol. 2017 Aug 23;13(8):937. doi: 10.15252/msb.20177548 (PMC5572396; doi:10.15252/msb.20177548)
Supplement: Supplementary file 2 — Expanded View Figures PDF [file MSB-13-937-s002.pdf]

Expanded View Figures

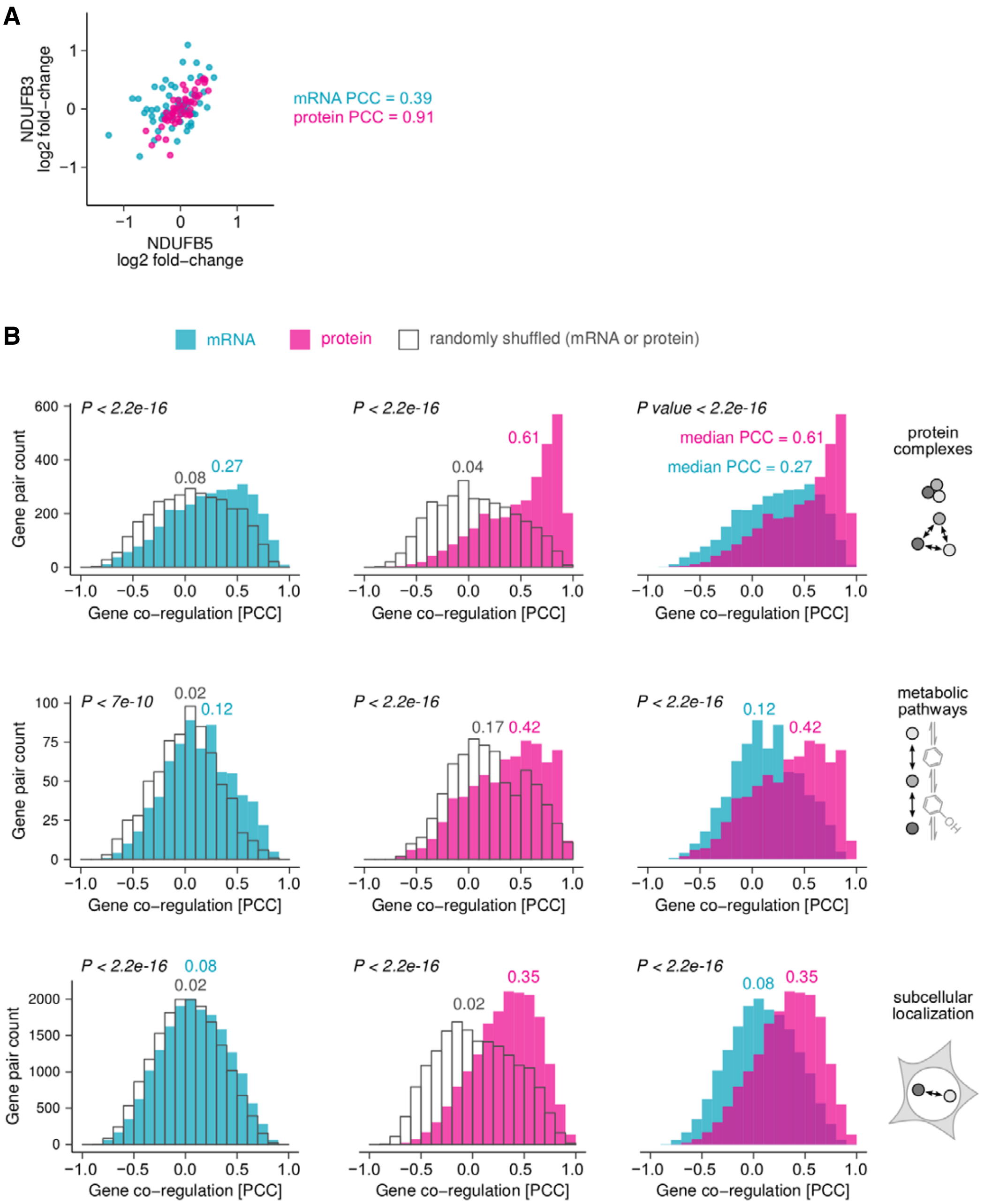

Figure EV1. Functionally related genes are co-regulated on mRNA level and, more strongly, on protein level.

A Expression levels of functionally related genes vary in a coordinated manner between LCLs, as measured using Pearson's correlation coefficient (PCC). Shown is the example of two genes encoding subunits of mitochondrial complex I. A PCC of 1 indicates perfectly correlated expression changes, 0 means no correlation and −1 indicates that changes are perfectly anti-correlated.

B We analysed gene pairs encoding subunits of the same protein complex, enzymes catalysing consecutive metabolic reactions and proteins with identical subcellular localisations. Histograms show the distributions of PCCs obtained for mRNA (turquoise) and protein (magenta) expression levels. As a control, we randomly shuffled each set of gene pairs (grey). Numbers above distributions indicate their median PCC. *P*-values from the Kolmogorov–Smirnov test show that the difference between test and control distributions, or mRNA and protein distributions, was highly significant. In all cases, protein abundance changes are significantly better correlated than the underlying mRNA changes.

© 2017 The Authors

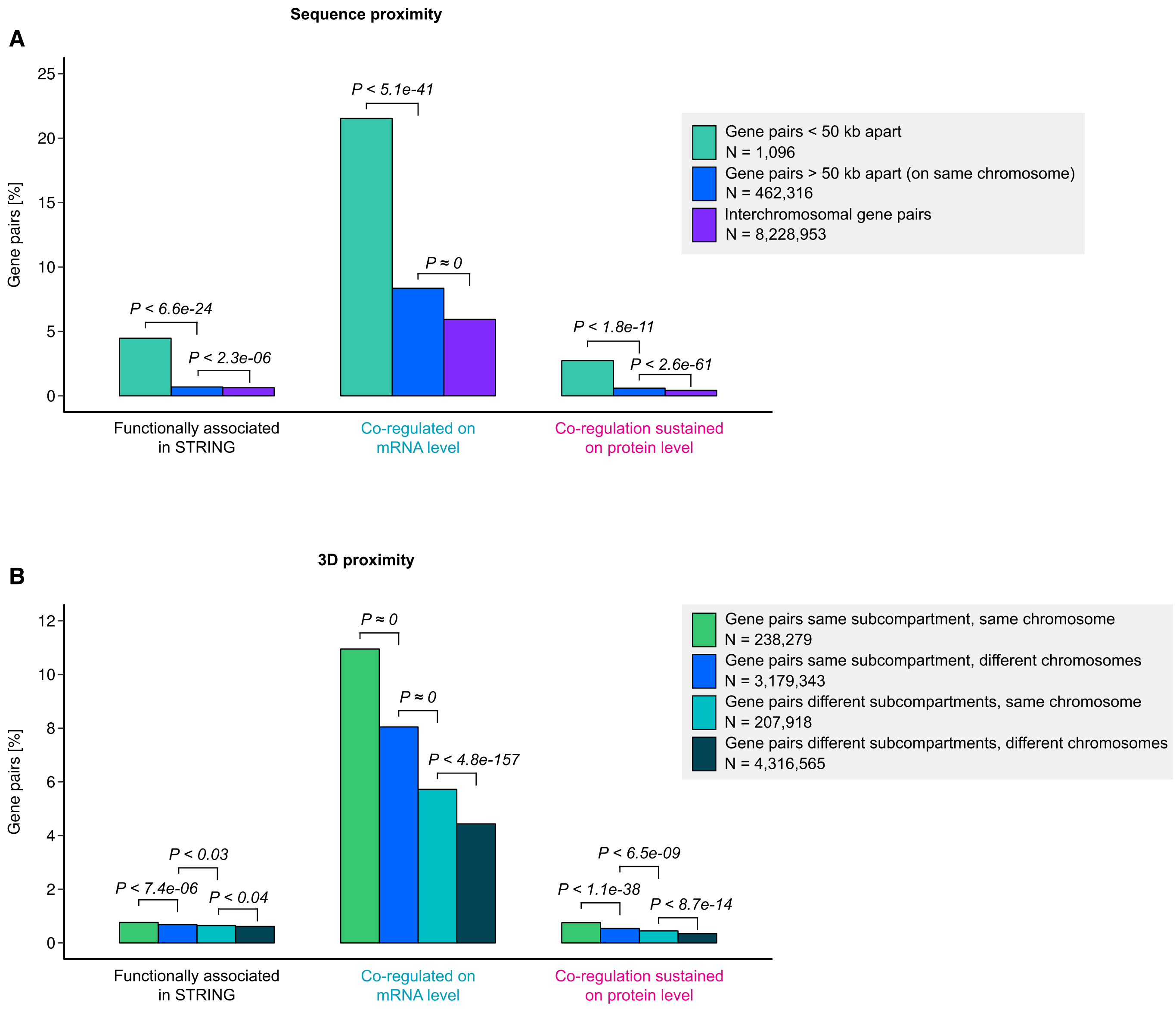

Figure EV2. Functionally related genes are enriched among nearby gene pairs, but more mRNAs are co-regulated than do co-function.

A Gene pairs with transcription start sites < 50 kb apart are enriched for genes with similar functions, relative to other genes on the same chromosome. Likewise, intrachromosomal gene pairs are enriched for similar functions relative to interchromosomal ones. However, the extent of mRNA co-regulation strongly exceeds co-function, and for most gene pairs, mRNA co-regulation is not sustained at the protein level. Functional associations were defined using STRING (Szklarczyk *et al*, 2017).

B Same as (A) but based on spatial proximity in 3D. Genomic subcompartments mapped by Hi-C experiments (Rao *et al*, 2014) are also enriched in genes with related functions, especially those belonging to the same chromosome. However, in quantitative terms, the increase of genes with similar function in subcompartments is marginal. As for linear proximity, for most closeby gene pairs with co-regulated mRNA abundances, co-regulation is not sustained at the protein level.

Data information: Gene pairs were defined as co-regulated based on a Pearson's correlation coefficient > 0.5 (BH-adjusted *P*-value < 0.001). *P*-values indicating differences between gene groups (bars) result from one-sided Fisher's exact tests.

 © 2017 The Authors

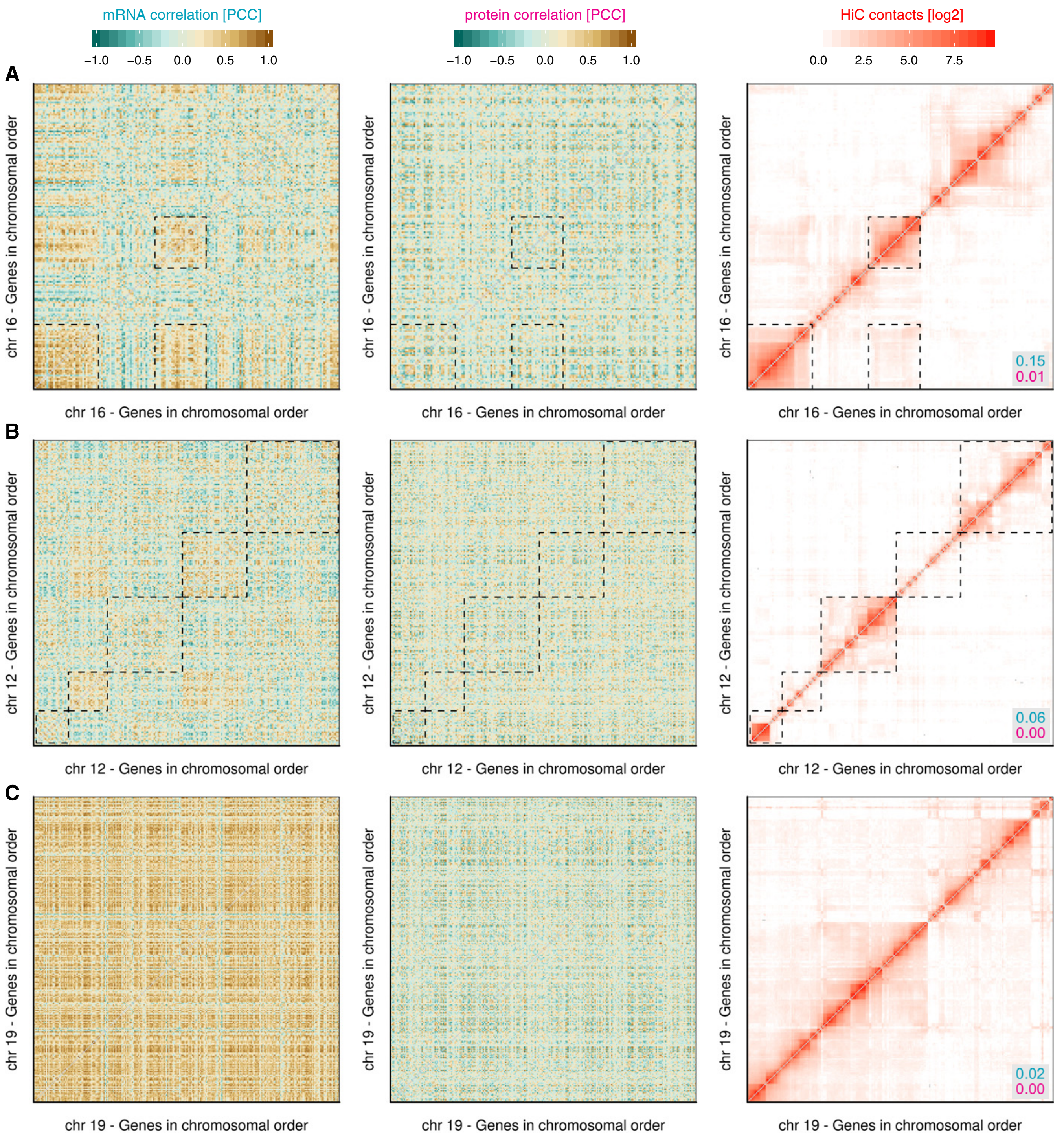

Figure EV3. Examples for intrachromosomal mRNA co-regulation patches.

A Chromosome 16 has several prominent patches of genes with strongly co-regulated mRNAs, similar to chromosome 11 shown in Fig 1D. These genes are not co-regulated on the protein level but interact with each other in the 3D structure of the genome. Note that the top right region displays increased Hi-C contact frequencies, but this is not reflected in the co-regulation map.

B Chromosome 12 is an example where only weak co-regulated patches are visible, and these align only partially with Hi-C contact patches.

C Chromosome 19 is a unique case, being a small but very gene-dense chromosome that is characterised by general co-regulation of most of its genes on the mRNA level, and a generally elevated Hi-C contact frequency, with no impact on protein co-regulation.

Data information: mRNA and protein co-regulation shown as Pearson's correlation coefficient (PCC). Dashed rectangles are shown for orientation. Numbers in grey box show the Pearson correlation between the Hi-C map and mRNA (blue) or protein (red) co-regulation maps.

© 2017 The Authors

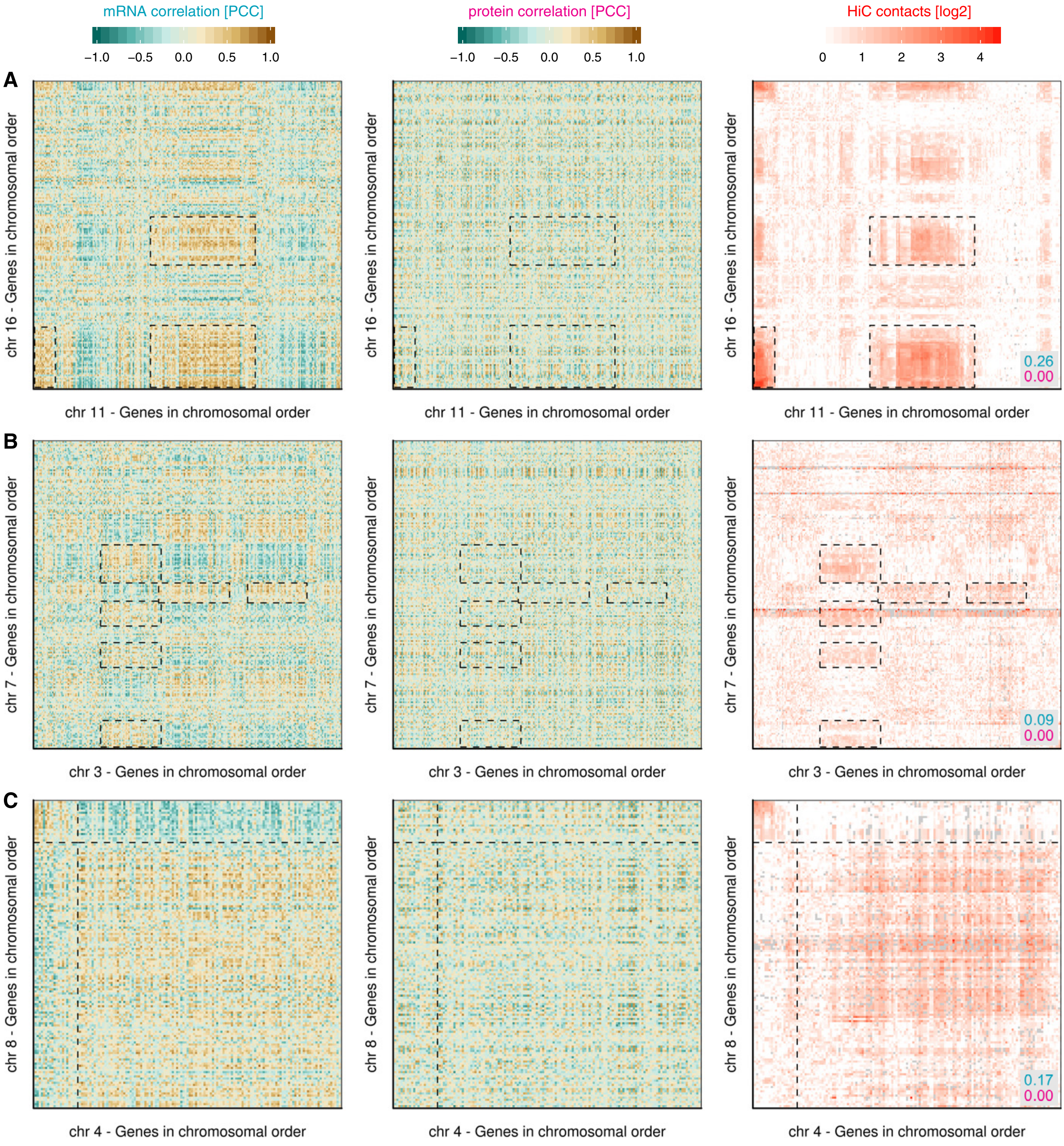

Figure EV4. Examples for interchromosomal mRNA co-regulation patches.

A Interchromosomal interactions between chromosomes 11 and 16. Multiple patches of genes with co-regulated mRNAs can be observed. These genes are not co-regulated on the protein level but interact with each other in the 3D structure of the genome.

B Similar co-regulation behaviour can be seen between genes from chromosomes 3 and 7, but in smaller patches.

C Between chromosomes 4 and 8 there is one very large patch of co-regulated genes, spanning most of each chromosome. These regions also tend to form Hi-C contacts with each other.

Data information: mRNA and protein co-regulation shown as Pearson's correlation coefficient (PCC). Dashed rectangles highlight co-regulated patches for orientation. Numbers in grey box show the Pearson correlation between the Hi-C map and mRNA (blue) or protein (red) co-regulation maps.

© 2017 The Authors
